# Lack of immunity against rubella among Italian young adults

**DOI:** 10.1186/s12879-017-2295-y

**Published:** 2017-03-07

**Authors:** Gallone Maria Serena, Gallone Maria Filomena, Larocca Angela Maria Vittoria, Germinario Cinzia, Tafuri Silvio

**Affiliations:** 1 Department of Biomedical Science and Human Oncology, University of Bari Aldo Moro, Piazza Giulio Cesare 11, 70124 Bari, Italy; 2 Hygiene Unit, Bari Policlinico General Hospital, Bari, Italy

**Keywords:** Rubella immunity status, Rubella elimination, Vaccination strategy, Serosurvey

## Abstract

**Background:**

To support the evaluation of the 2010-15 National Plan for Measles and Congenital Rubella Elimination, the authors designed and performed a serosurveillance survey to verify the immunity/susceptibility rate against rubella among Apulian young adults.

**Methods:**

The study was carried out from May 2011 to June 2012 in the Department of Transfusion Medicine/Blood Bank of Policlinico General Hospital in Bari. Subjects were enrolled by a convenience sampling. For each enrolled patient a 5 ml serum sample was collected and tested for anti-rubella IgG. The geometrical means (GMT) of anti-rubella IgG was calculated. T student test or ANOVA test, when appropriate, was used to compare the means of age per gender and GMT of anti-rubella IgG titres per age classes. Chi-square test was used to compare the proportion of anti-rubella IgG positive subjects per gender and per age classes. For all tests, a p value <0.05 was considered as significant.

**Results:**

At the end of the study 1764 subjects were enrolled, 1362 (77.2%) of which were male. The mean age was 38.4 ± 11.7 years (range: 17-65). 86.7% (95% CI = 85.0-88.2) had a positive titre of anti-rubella IgG. GMT of anti-rubella IgG titre was 4.3.

The proportion of positive subjects was of 76.8% (*n* = 279/363; 95% CI = 72.2-81.1) in persons aged 18-26 years; 88.1% (*n* = 310/352; 95% CI = 84.2-91.3) in 27-35 year-old people; 88.5% (*n* = 464/524; 95% CI = 85.5-91.1) in 36-45 year-old people; 90.7% (*n* = 350/386; 95% CI = 87.3-93.4) among people aged 46-55 years and 90.6% (*n* = 126/139; 95% CI = 84.5-94.9) in 55-65 year-old people (Chi-square = 39.7; *p* < 0.0001). GMT of anti-rubella IgG titre was 4.3 (4.3 in male and 4.2 in female, *t* = 2.2; *p* = 0.03) and seems to differ dividing the enrolled subjects by age group (*F* = 14.3; *p* < 0.0001).

**Conclusions:**

According to our data, too many women of child-bearing age are still unprotected from rubella in the elimination era and in this scenario the public health efforts should be oriented to catch-up activities.

## Background

The control of rubella is actually a global public health issue, because rubella virus infection during pregnancy may lead to fetal death or premature delivery, or may cause Congenital Rubella Syndrome (CRS), a condition which can involve all fetal organs, resulting in cataracts, deafness, heart defects, mental retardation. Congenital infections from rubella virus accounted for more than 100,000 cases worldwide in 1996, especially in countries with high rates of susceptibility to rubella among women of childbearing age [1].

A live-attenuated vaccine against rubella is available since 1960 [2] and in 2003 WHO reported that 131/215 countries/territories included rubella vaccine to their national immunization schedule. In 2002 two WHO regions, the Americas and Europe, established regional targets for elimination of rubella and CRS by the year 2010 [3–6].

A 2005–2010 strategic plan for eliminating measles and rubella and preventing CRS was launched by WHO European Region and within December 2012 all European countries introduced rubella-containing vaccines in their routine immunization programs; despite these public health efforts rubella is still circulating in Europe. The European Region number of rubella cases decreased from 804,567 in 1999 to 10,448 in 2010. Between 1990 and 2010, 467 cases of CRS were reported by 24 member states [7].

In Italy, the goal of rubella elimination and CRS prevention by year 2007 was first defined in the National Plan of Measles and Congenital Rubella Elimination 2003–2007 (PNEMoRC). Because the 2007 goal of elimination was not reached, the new PNEMoRC 2010–2015 deferred its achievement to the year 2015 and highlighted the need to strengthen the surveillance and reduce to less than 5% the susceptibility rate to rubella among women in childbearing age [8, 9]. After the PNEMoRC adoption, the vaccination coverage for rubella progressively increased; the 2013 national coverage assessed in children aged 24 months was 88% [10]. Immunization coverage of adolescents and adults is not routinely measured in Italy but, according to an epi-cluster survey conducted on 2008 in 18 of the 21 Italian regions, rubella vaccination coverage was 75% in 16 year-old adolescents [11].

The goal to reduce susceptibility rate to rubella to less than 5% among women in childbearing age has not been achieved yet and from 2005 to 2013 a total of 75 congenital rubella infections were reported in Italy; the national annual mean incidence was 1.5/100,000 live births [12].

Apulia is a region of the South of Italy with about 4 millions of inhabitants; according to PNEMoRc, in 2003 in Apulia an active offer strategy of rubella vaccination (using measles-mumps-rubella MMR vaccine) was implemented and immunization coverage progressively increased reaching 90%, but did not achieve the target coverage (95%) established by PNEMoRC [13]. From 2003 to 2011, one CRS, two confirmed and four suspected congenital infections and seven cases of rubella in pregnancy were observed in Apulia [14].

To support the evaluation of the 2010-15 PNEMoRC, the authors designed a seroprevalence survey to test the immunity/susceptibility rate against rubella among Apulian young adults. Serological surveillance is an important tool for the evaluation of vaccination programs as it monitors immunity status in the population and provides useful information to identify additional control measures, in order to the possible adoption of a third PNEMoRC. Moreover serological surveillance data are supplemental to coverage data and useful to compensate for many limitations of passive disease reporting systems for rubella. Passive reporting systems are often unreliable due to under notification of clinical cases and under diagnosis of subclinical cases which are estimated to be up to 50% of cases [15].

## Methods

The study was carried out from May 2011 to June 2012 in the Department of Transfusion Medicine/Blood Bank of Policlinico General Hospital in Bari.

Subjects were enrolled by a convenience sampling. A physician explained the aim of the study to blood donors; written informed consent was requested and obtained from blood donors who accepted to participate in the survey. The protocol of the study has been approved by the Regional Committee for the Epidemiology (Osservatorio Epidemiologico Regione Puglia). In accordance with Apulian Regional Laws, permission from the Ethics Commitee to carry out this study was not necessary given that both data and sera from patients were collected for routine diagnostic testing. The research was carried out in accordance with the Helsinky declaration.

Sample size was established considering the 2012 distribution of Apulian population by age groups (18-26; 27-35; 36-45; 46-55; 56-65) [16], using seroprevalence data from the first 100 enrolled subjects with a margin of error of 5% and a confidence interval of 95%. Age ranges were established in relation to the target cohort of MMR vaccination strategies (universal mass vaccination of all new-borns; catch-up strategies); older subjects were also enrolled to compare the sero-epidemiology of rubella in pre and post vaccination era.

For each enrolled patient we collected a 5 ml serum sample. All collected sera were tested for Anti-rubella virus IgG using the LIAISON® Rubella IgG chemiluminescence immunoassay (CLIA), a quantitative method. This is an indirect test performed with a standardized commercial method (DiaSorin, Saluggia, Italy) [17].

The samples with a concentration > 11 IU/mL are considered reactive (positive). In case of test with a borderline value, the test was repeated.

For each enrolled subjects, we completed a standardized form reporting age, gender, results of laboratory test. Forms were computerized using a database created by FileMaker Pro and data were analysed by STATA MP11.

We calculated the geometrical means (GMT) of anti-rubella IgG. T student test or ANOVA test, when appropriate, was used to compare the means of age per gender and GMT of anti-rubella IgG titres per age classes. Chi-square test was used to compare the proportion of anti-rubella IgG positive subjects per gender and per age classes. Linear regression was used to verify the correlation between age and anti-rubella IgG titre. For all tests, a p value <0.05 was considered as significant.

## Results

At the end of the study 1764 subjects were enrolled, 1362 (77.2%) of which were male. The mean age was 38.4 ± 11.7 years (range: 17-65), higher in male (39.4 ± 11.4) than in female (35.0 ± 12.1; *t* = 6.69; *p* < 0.0001). Table 1 shows the distribution of enrolled subjects by age group and gender.

**Table 1:** Distribution of enrolled subjects by age group and gender

Age group	Female		Male		Total	
	n	%	n	%	n	%
18-26	137	34.1	226	16.6	363	20.5
27-35	81	20.2	271	19.9	352	20.0
36-45	85	21.1	439	32.2	524	29.7
46-55	82	20.4	304	22.3	386	21.9
56-65	17	4.2	122	9	139	7.9
Total	402		1362		1764	

*Chi-square = 69.8; p < 0.001*

86.7% (95% CI = 85.0-88.2) of enrolled subject presented a titre of anti-rubella IgG >11 IU/mL. This percentage was higher in males (*n* = 1193/1362; 87.6%; 95% CI = 85.7-89.3) than in females (*n* = 336/402; 83.6%; 95% CI = 79.6-87.1; chi-square = 4.3; *p* = 0.04).

**Fig. 1:**
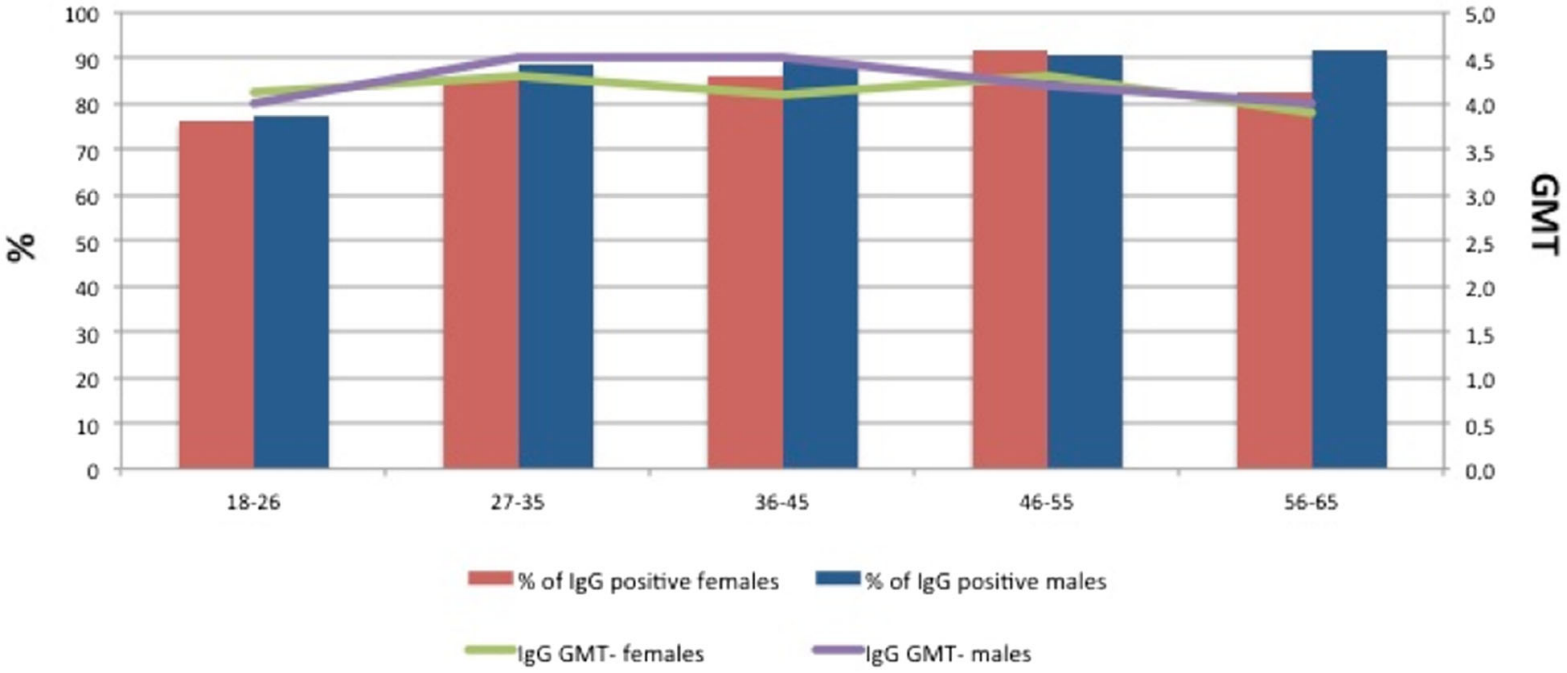
Proportion of anti-rubella IgG positive subjects and anti-rubella IgG GMT, by age group and gender. *Red columns*: % of anti-rubella IgG positive females. *Blue columns*: % of anti-rubella IgG positive males. *Green line*: anti-rubella IgG GMT in females. *Purple line*: anti-rubella IgG GMT in males

The proportion of positive subjects was of 76.8% (*n* = 279/363; 95% CI = 72.2-81.1) in persons aged 18-26 years; 88.1% (*n* = 310/352; 95% CI = 84.2-91.3) in 27-35 year-old people; 88.5% (*n* = 464/524; 95% CI = 85.5-91.1) in 36-45 year-old people; 90.7% (*n* = 350/386; 95% CI = 87.3-93.4) among people aged 46-55 years and 90.6% (*n* = 126/139; 95% CI = 84.5-94.9) in 55-65 year-old people (Chi-square = 39.7; *p* < 0.0001; Fig. 1).

GMT of anti-rubella IgG titre was 4.3 (4.3 in male and 4.2 in female, *t* = 2.2; *p* = 0.03) and seems to differ dividing the enrolled subjects by age group (Fig. 1; *F* = 14.3; *p* < 0.0001). There is no correlation between age and anti-rubella IgG titre (*r2* = 0.0006; *F* = 0.02; *p* = 0.87).

## Discussion

Our study showed a lack of immunity against rubella among Apulian young adults from 18 to 26 years of age; in this age group the proportion of susceptible subjects is more than 20%, in the other age groups the rate accounted 10-12%. The subjects with lack of immunity were born between 1988 and 1994, but only people born between 1990 and 1996 were a target of the 2003/2004 catch-up vaccination campaign planned by 2003 PNEMoRC.

A great proportion of young individuals showed lower levels of protective immunity, probably because they missed out on vaccination in catch up programmes. In fact, current coverage data for Apulian catch up target cohorts were around 70% [14]. This coverage determined a reduction in the circulation of wild-type infection, that could cause a lack of immunity among young adults and an increase of median age of infection.

PNEMoRC also recommended the free of charge offer of MMR vaccine to all susceptible females in childbearing age, but no specific actions for the active offer took place in Italian regions. The vaccination strategy based on passive offer seemed not consistent with the PNEMORC objective to reduce the percentage of susceptible females <5% [12].

Considering all enrolled females in childbearing age (18-49 years), the percentages of immune subjects is of 82.3% (95% CI = 77.9-86.2).

Our results quite differ from other studies carried out in countries that implemented Universal Mass Vaccination Program. In Australia, a serosurvey performed in 2001 (3 years after the beginning of Australian Measles Control Campaign) showed that the percentage of 16-39 years old subjects immune for rubella was 97% [18]. A similar rate was reported in the Netherlands where the rubella vaccine was actively offered to 11-years adolescents since 1974 and in 1987 the MMR vaccination was implemented in the national immunization program. In fact a 2014 survey showed a seroimmunity rate >90% among subjects aged >18 years [19]. In these countries national immunization campaigns reached target immunization coverage.

Our regional situation seems to remind the situation of USA in 1988-1994, when a serosurveillance study reported a rubella susceptibility rate of 22% among subjects of 18-24 years old [20]. Similarly to USA data, our results indicate the interaction between the immunization program and the natural history of rubella. In fact, in USA the rubella immunization program began in 1971 while in Italy in 2003, both the two studies were carried out 10-15 years after the beginning of immunization programs and results are globally concordant.

Bechini et al. carried out a serosurvey about rubella in Tuscany in 2012 and seroprevalence in this study (that involved a bigger number of females) is higher than in our survey, achieving 90%; this difference could be related to the enrolment of younger subjects (<18 years), that we did not consider in our sample [21].

The lesson learned is that in the elimination era too many women of childbearing age are still unprotected from rubella and this is consistent with the observation of some cases of CRS in Apulia in the last few years [14]. This could be related to low vaccine acceptance, which is lower mostly in the first years after the introduction of new vaccines, to insufficient management of vaccination strategies and to inappropriate perception by general population and health care workers of the risk of vaccine-preventable diseases [22].

In this scenario, the public health efforts should be oriented to catch-up activities in order to reduce the rate of susceptible young adults, above all women of childbearing age. Gynaecologists and General Practitioners should be encouraged to actively propose the rubella screening among women of childbearing age before they become pregnant, in order to identify those who lack rubella antibodies, whether acquired as the result of vaccination or natural infection.

The childbearing age susceptible women immunization is indeed a priority to be pursued in all possible occasions, especially in the postpartum days and for immigrant women. Rubella vaccination should also be administered in the hospitals before the patient discharge and the recommendation to get the rubella vaccination should be reported in the dismissal letter when it is not possible to vaccine during the hospitalization. Another opportunity to check rubella immunity status of the mothers is the access to the vaccination service for their own child immunization and health care workers should be encouraged to suggest mothers to investigate their immunity status during the pre-vaccination interview.

Finally an active surveillance based on laboratories that perform rubella immunity test should be planned; laboratories should notify to Public Health Authority every woman in childbearing age with a negative test and Public Health Authority should active propose to these women the immunization against rubella.

Future studies should investigate the effectiveness of this actions to reduce the rubella susceptibility rate among young women and decrease CRS, theorized in national and international recommendations, but actually not largely implemented.

## Conclusion

In conclusion, our sero-survey showed a lack of immunity against rubella among Apulian young adults from 18 to 26 years of age. According to our data, too many women of child-bearing age are still unprotected from rubella in the elimination era and in this scenario the public health efforts should be oriented to catch-up activities.

## Abbreviations

CRS: Congenital rubella syndrome
GMT: Geometrical mean titre
PNEMoRC: National plan of measles and congenital rubella elimination
RLU: Relative light units
RuV: Rubella virus
