# Erratum to: lack of immunity against rubella among Italian young adults

**DOI:** 10.1186/s12879-017-2724-y

**Published:** 2017-09-19

**Authors:** Maria Serena Gallone, Maria Filomena Gallone, Angela Maria Vittoria Larocca, Cinzia Germinario, Silvio Tafuri

**Affiliations:** 1 Department of Biomedical Science and Human Oncology, University of Bari Aldo Moro, Piazza Giulio Cesare 11, 70124 Bari, Italy; 2 Hygiene Unit, Bari Policlinico General Hospital, Bari, Italy

## Erratum

After publication of this article [1], the authors noted that the given names and family names of all authors had been inverted, and are therefore incorrect in the original article.

In the original article, the author names appear as the following:

Gallone Maria Serena, Gallone Maria Filomena, Larocca Angela Maria Vittoria, Germinario Cinzia and Tafuri Silvio.

However, this is incorrect, and the author names should appear as per the below:

Maria Serena Gallone, Maria Filomena Gallone, Angela Maria Vittoria Larocca, Cinzia Germinario, Silvio Tafuri.

The author names have been corrected in the author list and the citation for this Erratum.
